# Visual Outcome of Plasma Exchange in Optic Neuritis Associated With Flu Vaccination: A Case Report

**DOI:** 10.7759/cureus.30365

**Published:** 2022-10-16

**Authors:** Philip Bouchette, Maamannan Venkataraj, Natalie Jurisch, Pramod Kumar Ponna, Vishal Devarkonda

**Affiliations:** 1 Internal Medicine, Louisiana State University Health Sciences Center, Shreveport, USA

**Keywords:** systemic steroids, steroids, therapeutic plasmapheresis, flu vaccine, bilateral optic neuritis

## Abstract

Post-flu-vaccination optic neuritis is an extremely rare condition with an incidence ranging from 0.003 cases to 0.89 per 100 000 population. The exact pathophysiology is not clearly defined. Most of the patients with post-flu-vaccination optic neuritis tend to present with progressive worsening of vision in 2-3 weeks post-vaccine administration. A prompt fundus examination supplemented with MRI imaging of the orbit is required to establish the diagnosis. On diagnosis, early initiation of high-dose oral or IV steroids is recommended to prevent optic atrophy or worsening of vision. Most patients tend to have complete recovery of vision when started on steroids. However, if the patient continues to have worsening symptoms while being treated with a high dose of steroids, plasmapheresis (PLEX) is an effective intervention.

## Introduction

Post-flu-vaccination optic neuritis is an extremely rare condition with an incidence ranging from 0.003 cases to 0.89 per 100 000 based on different population studies. Vaccine-associated optic neuritis is based on the temporal relationship between the administration of vaccines and the development of optic neuritis in the absence of other etiologies. The exact pathophysiology is not clearly defined. Patients typically develop symptoms 2-3 weeks post administration of vaccine and with good recovery in visual acuity when treated with steroids. We present a rare case of optic neuritis after receiving inactivated flu vaccination with no significant improvement with high-dose steroids initially, followed by recovery in visual acuity when started on plasmapheresis (PLEX).

## Case presentation

A 63-year-old female with a past medical history of type II diabetes mellitus and hypertension initially presented to an outside facility with a persistent headache and blurring of vision for over two weeks. She endorsed that the symptoms were associated with rhinorrhea, coughing, sore throat, myalgias, diarrhea, and nausea at the onset of headache; however, these symptoms had resolved at the time of admission. On the day of presentation, the patient reported that she could not read text messages prompting her to seek evaluation at the hospital. The patient reported receiving flu vaccination three weeks before the hospital encounter. She denied any fever, dizziness, lightheadedness, or focal neurologic deficits. Her primary care physician treated her for possible upper respiratory tract infection with oral antibiotics and antihistamines one week before admission.

On physical examination, the patient was found to have a bilateral decrease in visual acuity with no other focal neurological deficits. Visual acuity in right eye: finger count 2’ without correction and left eye: 20/200 -1 with correction. The patient's blood chemistry was negative for infective or metabolic abnormalities. Initial CT scan of the brain was negative for the acute process. Nasopharyngeal swab for influenza was negative. Lumbar puncture with cerebrospinal fluid (CSF) analysis showed a white cell count of 29 cells/cumm with more than 97% lymphocytes (Tables 1-2). CSF glucose was elevated at 80 mg/dL and CSF protein was within normal limits. Lyme serology in CSF and West Nile virus by polymerase chain reaction (PCR) in CSF were negative. OG-IgG1 screening was positive with a titer of 1:100.

**Table 1:** CSF cell count and differential CSF=cerebrospinal fluid; WBC=White blood cell; RBC=Red blood cell; Lymphs= Lymphocytes; Cu mm=Cubic millimeters

Heme Aliquot	mL	1.0
Appearance, CSF	Clear	Clear
Color, CSF	Colorless	Colorless
WBC, CSF	0 - 5/cu mm	29/cu mm
RBC, CSF	0/cu mm	0/cu mm
Lymph’s, CSF	40 - 80%	97%
Mono/Macrophage, CSF	15 - 45%	3%

**Table 2:** CSF results CSF=cerebrospinal fluid; WBC=White blood cell; RBC=Red blood cell; Lymphs= Lymphocytes; Ab=Antibody; CNS=Central Nervous System; IgG=ImmunoglobulinG; Mg/dl=milligram/deciliter

TEST NAME	Reference range	Results
Glucose CSF	40-70 mg/dl	80 mg/dl
Protein CSF	15-40 mg/dl	36 mg/dl
Lyme Disease Serology, CSF	Negative	Negative
Lyme Disease Ab Interpretation	Negative	Negative
Lyme CNS Infection, IgG, Serum	Negative	Negative
West Nile Virus Result	Negative	Negative

**Figure 1:**
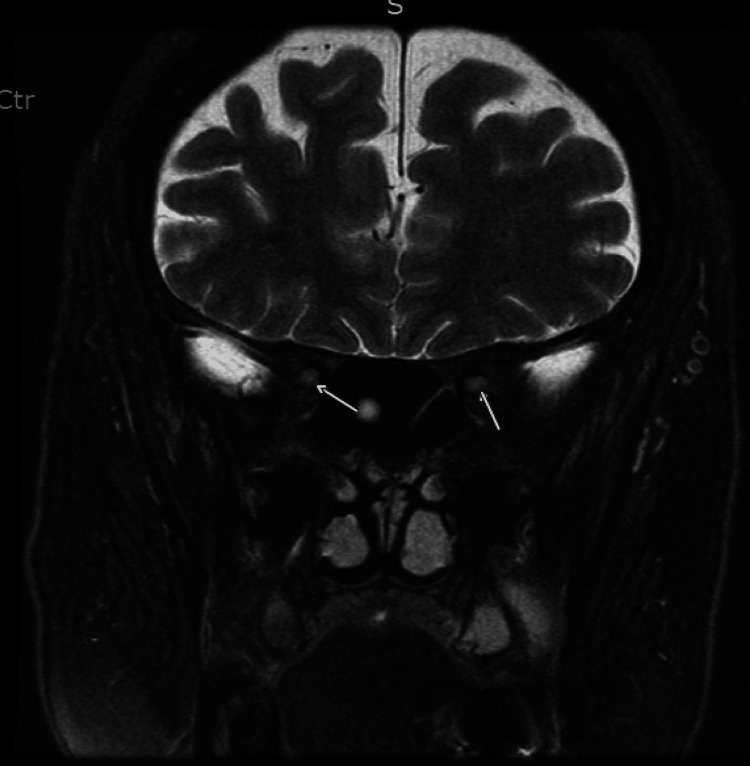
Findings - Orbits/optic nerves: no abnormal signal or enhancement of the right or left globe, preseptal soft tissues, intra-orbital soft tissues. Mild contrast enhancement of optic nerves, right more than left, seen on postcontrast images, subtle T2 hyperintensity near optic canal (white arrows). Remainder of the intracranial compartment: normal. Skull/extracranial contents (limited evaluation): bone marrow signal intensity normal. Impression: subtle enhancement of optic nerves, right more than left, suggestive of optic neuritis.

The ophthalmology team was consulted and recommended MRI orbits that revealed subtle enhancement of the bilateral optic nerves, right-sided/greater than left, suggestive of bilateral optic neuritis possibly induced by the flu vaccine (Figure 1). The patient was started on 1 g Solu-Medrol daily for five days and was discharged on 80 mg of oral prednisone. The patient was subsequently followed in the ophthalmology clinic the next day after discharge. The patient reported having continued loss of vision with visual acuity (Snellen-Linear) in the right eye being positive for hand movement without correction and finger count at 3 feet with correction. Fundus examination in the right eye showed 3+ edema, flame, and heme deposit, and the left eye showed 2+ pedal edema. Since there was no significant improvement in the patient's visual acuity on high-dose steroids, the patient was transferred to our facility for PLEX. She received five sessions of PLEX while being maintained on steroids with gradual improvement in her visual acuity. The patient was discharged on oral steroids with outpatient follow-up with ophthalmology.

## Discussion

This is a rare case of a patient with bilateral optic neuritis who developed visual disturbance 2-3 weeks post-inactivated flu vaccination, which was initially refractory to high-dose intravenous and oral steroids, followed by significant improvement in vision after undergoing PLEX. Jun et al., in 2018, reviewed the literature and reported 10 familiar cases of unilateral and bilateral optic neuritis following flu vaccination [1]. Among the 10 cases, they reported that seven patients were males. Partial or complete recovery was reported in seven out of eight patients receiving IV or oral steroids, with one patient having no improvement in vision. Two of the ten patients who did not receive any form of steroids were found to develop optic atrophy in the future. These cases reported in the literature should not be considered proof of the cause. Alteration of immune activity following flu-vaccine administration could possibly be linked to optic neuritis and another autoimmune/autoinflammatory disease.

In 2011, Shoenfeld et al. coined the term autoimmune/inflammatory syndrome induced by adjuvants (ASIA). Adjuvants denote several substances that drive innate immunity and boost immune activity against the antigens [2]. Watad et al., in 2019, evaluated data from the ASIA registry and performed an analysis of 500 cases. Their study of 500 patients showed that the mean age of having ASIA following vaccine administration was 43±17 years, with primarily female predisposition at 89% [3]. Well-defined autoimmune diseases contribute to 69% of these cases. However, in the United States, most vaccines, including polio, rotavirus, chickenpox, seasonal influenza, measles, mumps, and rubella, do not contain added adjuvants [4]. In 2015, the US Food and Drug Administration (FDA) approved the first seasonal quadrivalent influenza vaccine containing an adjuvant called FLUAD for the elderly population above 65 years to create a more robust immune response [5]. Observing the long-term data regarding optic neuritis/autoimmune/inflammatory syndrome associated with this vaccine would be interesting.

Given the lack of literature available on optic neuritis following the flu vaccine, the timing and role of PLEX in the management of optic neuritis are debatable. Skorupka et al., in 2019, performed a retrospective monocentric study to evaluate the visual outcomes of plasma exchange treatment of steroid-refractory optic neuritis and reported significant improvement in visual acuity [6]. The time interval from the onset of symptoms until treatment initiation with high-dose steroids was 8.4 (4.2-12.5) days and initiation of PLEX 20.3 (14.8-25.9). Early identification and initiation of high-dose IV or oral steroids and close monitoring of its visual acuity and fundus examination would be an acceptable initial approach.

## Conclusions

Given the constraints in resources while performing PLEX and the lack of trials comparing the benefits of high dose of steroids vs. early PLEX, early identification and initiation of high-dose IV or oral steroids and close monitoring of its visual acuity and fundus examination would be an acceptable initial approach. Physicians should consider PLEX in evaluating the patient's response to steroids, improvement in visual acuity, and fundus examinations. 

## References

[1] Jun B, Fraunfelder FW (2018). Atypical optic neuritis after inactivated influenza vaccination. Neuroophthalmology.

[2] Shoenfeld Y, Agmon-Levin N (2011). 'ASIA' - autoimmune/inflammatory syndrome induced by adjuvants. J Autoimmun.

[3] Watad A, Bragazzi NL, McGonagle D (2019). Autoimmune/inflammatory syndrome induced by adjuvants (ASIA) demonstrates distinct autoimmune and autoinflammatory disease associations according to the adjuvant subtype: Insights from an analysis of 500 cases. Clin Immunol.

[4] Sudarshan S, Huang EH, Lim PL, Leo YS, Lim SA (2012). A case of post-vaccination optic neuritis: coincidence or causative?. Eye (Lond).

[5] (2022). Adjuvanted flu vaccine. https://www.cdc.gov/flu/prevent/adjuvant.htm.

[6] Skorupka N, Miclea A, Jalowiec KA (2019). Visual outcomes of plasma exchange treatment of steroid-refractory optic neuritis: a retrospective monocentric analysis. Transfus Med Hemother.

